# Comprehensive survey on urological stent practices by the European Association of Urology (EAU) Endourology

**DOI:** 10.1007/s00345-026-06612-w

**Published:** 2026-07-19

**Authors:** Burak Akgul, Selcuk Guven, Gernot Ortner, Hakan Akdere, Bhaskar Somani, Vineet Gauhar, Steffi Kar Kei Yuen, Panagiotis Kallidonis, Thomas RW Herrmann, Udo Nagele, Theodoros Tokas

**Affiliations:** 1 Department of Urology, School of Medicine, Indiana University, Indianapolis, IN USA; 2 Department of Urology, Meram School of Medicine, Necmettin Erbakan University, Konya, Turkey; 3 Department of Urology and Andrology, Hall in Tirol, General Hospital Hall I.T., Hall in Tirol, Austria; 4 Training and Research in Urological Surgery and Technology (T.R.U.S.T.)-Group, Hall in Tirol, Austria; 5 Department of Urology, School of Medicine, Trakya University, Edirne, Turkey; 6 Department of Urology, University Hospital, Southampton NHS Trust, Southampton, UK; 7 Department of Urology, Ng Teng Fong General Hospital, Singapore, Singapore; 8 Department of Surgery, S.H. Ho Urology Centre, The Chinese University of Hong Kong, Hong Kong, China; 9 Department of Urology, University of Patras, Patras, Greece; 10 Department of Urology, Kantonspital Frauenfeld, Spital Thurgau AG, Frauenfeld, Switzerland; 11 Department of Urology, Medical School, University General Hospital of Heraklion, University of Crete, Heraklion, Greece

**Keywords:** Endourology, Ureteral stent, Practice variability, Stent-related symptoms

## Abstract

**Purpose:**

Ureteral stents are widely used following ureteroscopy (URS), yet several aspects of their management remain non-standardized despite contemporary EAU and AUA guideline recommendations. This study evaluated contemporary international practices and identified gaps in ureteral stent use among endourologists.

**Methods:**

A cross-sectional international survey consisting of 58 items across nine domains was distributed via social media, major urological meetings, and the EAU Endourology Section. Only fully completed responses were analyzed. Descriptive statistics were used, with continuous variables reported as medians and categorical variables as frequencies and percentages.

**Results:**

A total of 121 respondents from 48 countries were included. Stent placement remained common after uncomplicated ureteroscopy and was nearly universal in complicated cases. Pre-stenting was applied selectively, most commonly after failed ureteroscopy and for ureteral dilatation. A 6 Fr stent and polyurethane material were the preferred choices. Stent-related symptoms were frequent, with urgency and frequency predominating, followed by hematuria. Pharmacologic management varied, and conservative treatment was commonly used for post-stenting infections. Notably, 45% of respondents did not use a structured stent-tracking system and 71.8% had never used a validated symptom-assessment tool such as the USSQ. Exploratory subgroup analyses identified differences according to geographic region, institution type, and endourological training background in selected aspects of stent management, including pre-stenting duration, long-term stent exchange protocols, pharmacologic management, and follow-up systems.

**Conclusion:**

International ureteral stent practices show substantial variability. Although core practices were broadly comparable across regions, important gaps remain in stent tracking and symptom assessment, highlighting opportunities to improve patient safety and standardize care.

## Introduction

Ureteral stents are widely used in endourological practice, particularly following ureteroscopy (URS), but are associated with significant morbidity and variability in clinical management [1]. Stent-related symptoms affect up to 80% of patients and can substantially impair quality of life, while complications such as infection and encrustation remain important concerns, particularly with prolonged dwell time [2]. Despite advances in pharmacologic management and stent technology, symptom control remains suboptimal, and standardized assessment tools such as the Ureteral Stent Symptom Questionnaire (USSQ) are rarely used in routine practice [3]. The necessity of stenting after uncomplicated URS remains controversial. Although evidence suggests that routine stenting may be avoided in selected cases, real-world practice continues to demonstrate high utilization rates [4]. Contemporary EAU and AUA guidelines similarly recommend avoiding routine stent placement after uncomplicated URS in appropriately selected patients. In addition, optimal stent characteristics, including size, material, and duration, remain poorly defined [5]. Patient safety concerns, particularly related to delayed removal and inadequate follow-up systems, further highlight gaps in current practice. Therefore, this study aimed to evaluate contemporary international practices, preferences, and decision-making patterns in ureteral stent use among endourologists.

## Materials and methods

The questionnaire included single- and multiple-choice questions, as well as frequency-based questions, with certain items, particularly those related to clinical indications, allowing multiple responses to better reflect real-world decision-making, where more than one indication may coexist. It comprised 58 items organized into nine thematic domains, including participant characteristics (institution type, current role, and area of expertise), endourology training background, stenting indications and frequency, stent design and material preferences, duration and removal practices, special clinical scenarios, technical aspects, complications and symptom management, patient communication, and characteristics of the “ideal” ureteral stent. For subgroup analyses, respondents were categorized according to their endourological training background as fellowship-trained, primarily residency-trained, or having acquired their endourological expertise predominantly during independent consultant practice. Before dissemination, the questionnaire was reviewed, refined through three iterative sessions by BA, SG, BS, VG, and TT, and pilot-tested before finalization.

The survey was disseminated through multiple channels to maximize international reach and participation, including social media platforms and major international urological meetings such as the International Conference on Endourology, the European Association of Urology (EAU) Istanbul Pediatric Urolithiasis Meeting, the 68th Philippine Urological Association Global Urologic Summit, and the Algerian Community of Endourology and Laparoscopic Surgery Meeting. Additionally, it was circulated through the EAU Endourology Section network, ensuring broad engagement among endourology-focused urologists. The full questionnaire is provided as Supplementary Material. After 2 months, responses were collected, and a descriptive analysis was conducted. No incentives were provided to participants. Descriptive statistics were used to summarize the data. Only fully completed questionnaires were included in the final analysis; partially completed responses were excluded. Continuous variables were reported as median (interquartile range), and categorical variables as frequencies and percentages. In addition, exploratory subgroup analyses were performed according to geographic region (Europe, Asia-Pacific, North and South America, and the Middle East), institution type, and endourological training background. Associations between categorical variables were evaluated using Pearson’s chi-square test, and Linear-by-Linear Association testing was applied when appropriate for ordinal variables. Given the exploratory nature of these analyses, p values were interpreted descriptively. All statistical analyses were performed using SPSS version 31.0.

## Results

**Fig. 1:**
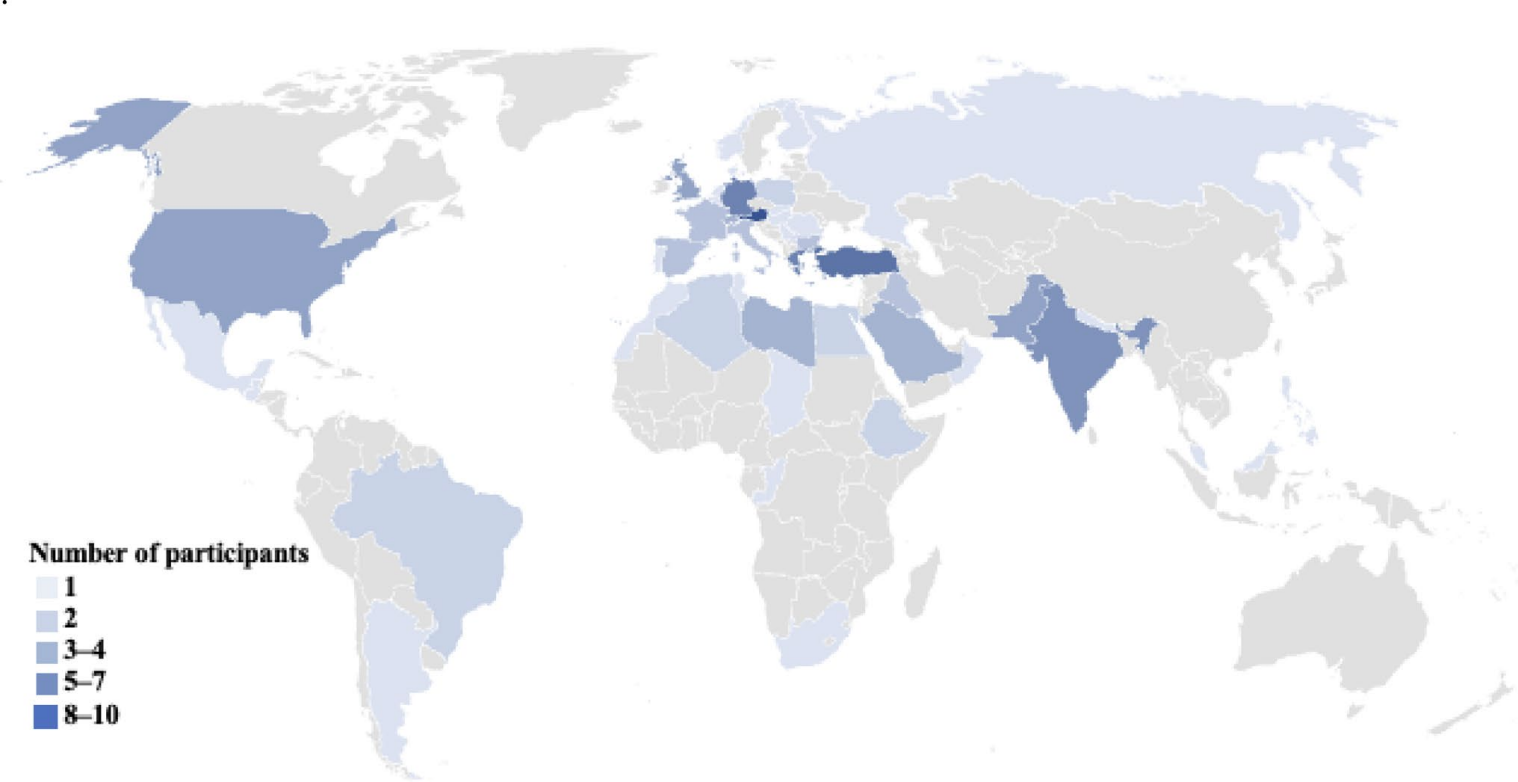
Illustrates the geographical distribution of participants

A total of 146 participants responded to the survey, of whom 121 fully completed the questionnaire and were included in the final analysis. Respondents represented 48 countries. The highest numbers of respondents were from Austria, Turkey, Germany, Greece, India, the United States, the United Kingdom, and Pakistan, respectively. These countries were followed by 40 other countries. The median age was 44 years (interquartile range 39–55), and the majority were male (93%). Most participants were affiliated with university hospitals (58%), followed by public (23%) and private hospitals (19%). The majority were senior surgeons or consultants (82%), followed by endourology fellows (10%) and urology residents (9%). Endourology–urolithiasis was the primary area of expertise for 84% of participants. Regarding endourological training background (n = 121), 42.0% of respondents received their primary endourological training during residency, 31.9% had completed a dedicated endourology fellowship, and 26.1% reported acquiring most of their endourological expertise during independent consultant practice. The country distribution is shown in Fig. 1.

### Stenting practices after URS

**Fig. 2:**
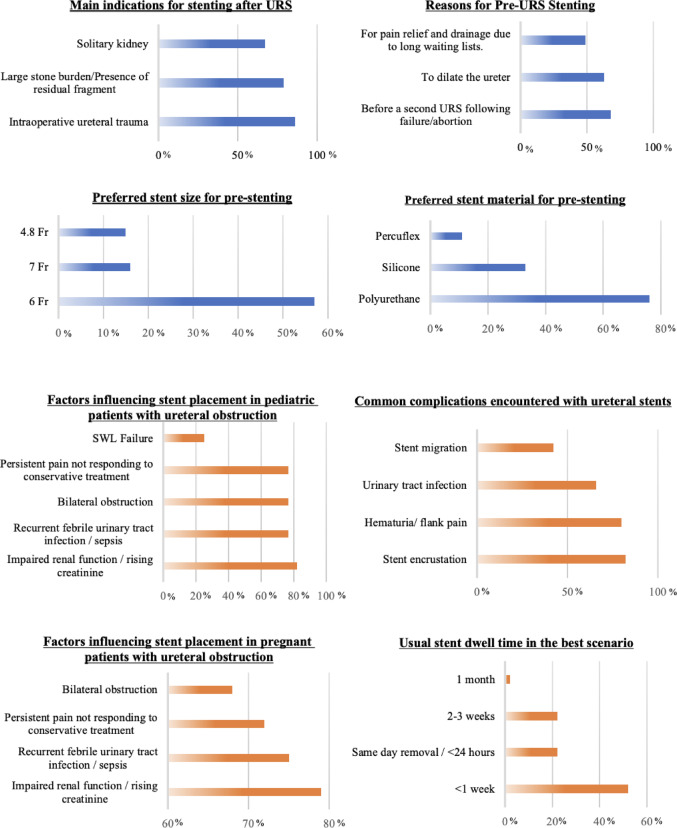
Distribution of responses across key clinical scenarios in international ureteral stent practice, demonstrating variability in clinical decision-making

Following uncomplicated URS, routine stent placement remained common among respondents. The most frequently reported indications included intraoperative ureteral trauma, large stone burden or residual fragments, and the presence of a solitary kidney (Fig. 2). In complicated cases, the tendency to place a ureteral stent was high, with 73.3% of respondents reporting that they always placed a stent.

### Stent characteristics

In routine clinical practice, 6 Fr was the most commonly used stent size, followed by 4.8 Fr and 7 Fr, and was also the preferred size for pre-stenting. Polyurethane was the predominant stent material, followed by silicone, while metallic, drug-eluting, and biodegradable stents were rarely used. The most commonly used stent length was 26 cm in male patients and 24 cm in female patients. Overall, 43% did not adjust stent length based on patient height, while 23% always did so; 15% adjusted it in less than half of cases, and 19% in more than half. Adjustment based on laterality was uncommon (82% did not). Most respondents had never used hydrogel-coated or drug-eluting stents, and their use remained below 5% in 25.9% of cases. Similarly, most had never used metallic stents, with 38.7% reporting use in less than 5% of cases. Awareness of biodegradable stents was low.

In the best-case scenario, following uncomplicated URS, 52% of respondents removed the stent within 1 week, 22% within 2–3 weeks, and 20% performed same-day removal. After complicated URS, half of the respondents reported stent removal within 2–3 weeks. Overall, 33% did not use stents with extraction strings, while 30% used them in less than 5% of cases and 25% in less than half of cases. Stents with extraction strings were most commonly preferred after uncomplicated URS, particularly when short-term stenting (< 1 week) was planned, as well as in patients with high anxiety or those unable to tolerate office-based endoscopic removal.

Most respondents exchanged long-term stents at 6-month intervals (60%), while 20% preferred 3-month intervals, and another 20% extended the interval to 12 months. Notably, 45% did not use any reminder or alert system to track stent removal, whereas only 39% consistently used structured alert systems. When a suction access sheath was used during URS, 62% of respondents reported postoperative stent placement always or in more than 50% of cases, whereas 29% reported stent placement in less than 50% of cases and 9% reported never placing a stent. Postoperative stenting was also common among users of suction-enabled ureteroscopes, with 63% reporting stent placement always or in more than half of cases. Overall, the variability across these clinical aspects is further summarized and illustrated in Fig. 2.

### Special clinical scenarios

In pediatric patients, ureteral stent placement was most commonly associated with clinical factors such as impaired renal function, infection, and obstructive burden, whereas procedural factors were less frequently reported. When intervention was required, ureteral stenting was most commonly preferred when a minimally invasive, temporary drainage approach was prioritized (77%). It was also frequently selected when rapid decompression was necessary (67%) and when URS was not feasible or contraindicated. Regarding stent characteristics, 4.8 Fr was the most commonly preferred stent size (45%), followed by 4 Fr (33%) and 3.7 Fr (22%). In terms of stent length, 22–26 cm was the most commonly selected option, although a notable proportion of respondents reported tailoring stent length according to patient characteristics. Polyurethane-based stents, particularly small-caliber variants, were among the most commonly preferred materials, followed by silicone stents.

In pregnant patients with ureteral obstruction, impaired renal function or rising creatinine emerged as the most influential factor for ureteral stent placement (~ 78%), followed by recurrent febrile urinary tract infection or sepsis (~ 75%) and persistent pain unresponsive to conservative management (~ 72%), while bilateral obstruction was a less frequently reported determinant (~ 68%).

When intervention was required, ureteral stenting was most commonly favored in settings requiring rapid decompression (75%) or when a minimally invasive, temporary drainage approach was preferred (73%). It was also commonly utilized as a bridging strategy to safely defer definitive treatment until the postpartum period (69%).

In ureteral strictures, 6 Fr stents were most commonly used (53.9%), followed by 7 Fr (26.9%). Polyurethane was the most frequently preferred material (32.2%), followed by silicone and metallic stents. In pediatric cases, smaller-caliber stents were favored, with 4.8 Fr being the most commonly used size, and stent length was generally tailored to the patient’s body size. Polyurethane also remained the most commonly used material in this group.

For recurrent or chronic infections, 45.7% preferred 6 Fr stents, with polyurethane as the predominant material, while 57.3% favored 6 Fr stents for long-term indwelling stenting. Fluoroscopic guidance was widely used (56.9% always, 19.8% in > 50% of cases). Routine confirmation of stent position with X-ray or ultrasound was also common, with 61.9% always performing it.

### Stent-related symptoms and management

Stent-related symptoms were common, occurring in 25–50% of cases in 38.5% of respondents and in 50–75% in 29.9%. Urgency (79.2%) and frequency (75.0%) were the most frequently reported symptoms, followed by hematuria (67.7%) and dysuria (66.7%), while flank pain (56.3%) was less common. Alpha-blockers or anticholinergics were used in more than half of cases by 35% of respondents, always by 15%, and in less than half of cases by 33%. Following ureteral stent placement, 74% managed patients presenting with fever conservatively, using antibiotics and close follow-up, while the remainder opted for stent exchange after initiating antibiotics and performing a sepsis workup.

### Follow-up and patient safety

The majority of respondents (71.8%) had never used standardized symptom assessment tools such as the USSQ. A dedicated stent follow-up protocol or clinic was present in 61.1%, while 38.9% reported not having such a system. Notably, the presence of a follow-up protocol did not necessarily imply the use of an electronic tracking mechanism, as 45% of respondents reported not using reminder or alert systems for stent tracking and removal. Written instructions were not provided by 41.6% of respondents, whereas 32.7% reported always providing them. The definition of an ideal stent most commonly includes comfort and minimal patient symptoms, followed by long indwelling time with resistance to encrustation, ease of insertion and removal, cost-effectiveness and availability, and adjustable or variable length options. Table 1 summarizes key findings and international practice patterns in ureteral stent use, highlighting major trends and their clinical interpretations.

**Table 1:** Summary of key findings and international practice patterns in ureteral stent use

Parameter	Key finding	Majority trend	Suggested interpretation
Post-URS stenting (Uncomplicated)	Stent placement frequently performed	37% (75–100% of cases) 21% (50–75% of cases)	Routine stenting remains common despite uncomplicated cases
Post-URS indications	Ureteral trauma, stone burden, solitary kidney	86% (ureteral trauma) 79% (stone burden) 67% (solitary kidney)	Risk-based stenting
Complicated URS	Stent placement nearly universal	73% (always)	Strong agreement for mandatory stenting
Pre-stenting practice	Not routinely performed	52% (no routine pre-stenting)	Selective use rather than routine
Pre-stenting indications	Previous failed URS, ureteral dilatation, pain and prolonged waiting time	65% (failed URS) 63% (ureteral dilatation) 49% (pain and prolonged waiting time)	Primarily used for technical facilitation and symptom control
Pre-stenting duration	Most common duration 1–2 weeks	57% (1–2 weeks duration)	Short-term pre-stenting preferred
Stent size (Pre-stenting and after URS)	6 Fr most commonly preferred	Most respondents	Standard stent size in clinical practice
Stent material	Polyurethane most commonly used, followed by silicone	Most respondents	First-line materials in routine practice
Ureteral strictures – stent size	6 Fr most commonly used, followed by 7 Fr	53.9% (6 Fr) 26.9% (7 Fr)	Preference for larger-caliber stents in strictures
Ureteral strictures – material	Polyurethane most preferred, followed by silicone and metallic	32.2% (polyurethane)	Conventional materials remain first-line
Pediatric cases Stent size	4.8 Fr most commonly used, followed by 4 Fr and 3.7 Fr	45% (4.8 Fr) 33% (4 Fr) 22% (3.7 Fr)	Smaller-caliber stents preferred
Pediatric cases Stent length	Individualized by patient body size	Most respondents	Tailored approach preferred over fixed sizing
Pediatric cases Stent material	Polyurethane most commonly used	Most respondents	Consistent material preference across populations
Stent-related symptom burden	Symptoms occur in 25–50% and 50–75% of cases	38% (symptoms in 25–50% of cases) 29% (symptoms in 50–75% of cases)	Stent-related symptoms are highly prevalent
Most common symptoms	Urgency and frequency most frequent	79% (urgency) 75% (frequency)	Bladder irritation is the predominant issue
Symptom assessment tools	Rarely used in clinical practice	71% (never use)	Limited adoption of standardized symptom assessment
Medical management after stent placement	Variable use of α-blockers and anticholinergics	35% (use in > 50% of cases) 15% (always)	No standardized pharmacologic approach
Fever management	Conservative treatment preferred	74% (conservative management)	Initial antibiotics and close follow-up favored
Second-line management	Stent exchange after antibiotics and sepsis workup	Minority of participants	Reserved for selected or severe cases
Follow-up	Dedicated stent follow-up protocol in place	61.1% (dedicated follow-up protocol in place)	Growing awareness of stent tracking importance
Tracking gaps	No structured follow-up system in place	38% (no structured follow-up system)	Significant patient safety gap
Patient instructions	Inconsistent provision of written instructions	41% (never) 32% (always)	Lack of standardized patient education

### Subgroup analyses according to geographic region, institution type, and endourological training background

When stratified by geographic region (Europe, Asia-Pacific, the Americas, and the Middle East), most stent-related practices were comparable across regions. Significant regional differences were identified only in the preferred stent diameter for ureteral strictures (χ²=35.36, *p* = 0.002) and the routine prescription of alpha-blockers or anticholinergics for stent-related symptoms (χ²=16.78, *p* = 0.048). Apart from these findings, regional practices were largely comparable, with no significant differences observed in postoperative stenting practices, pre-stenting strategies, stent dwell times, major stenting indications, stent tracking systems, or reported stent-related symptom burden.

Analysis according to institution type revealed significant differences in several aspects of ureteral stent management, including stent diameter selection in routine practice and pre-stenting, postoperative stenting after suction access sheath use, and the availability of dedicated stent follow-up protocols.

When stratified according to endourological training background, significant differences were observed in pre-stenting duration and long-term stent exchange protocols. Respondents with fellowship training more frequently preferred a 2-week pre-stenting duration, whereas residency-trained respondents more commonly favored shorter stent exchange intervals. Although routine imaging confirmation of stent position did not reach statistical significance, a significant linear trend was observed across training backgrounds. No significant differences were identified in the principal indications for postoperative stenting, pre-stenting, or stent placement in pediatric and pregnant patients according to institution type or endourological training background. The significant and trend-level differences identified according to geographic region, institution type, and endourological training background are summarized in Table 2.

**Table 2:** Summary of significant and trend-level differences according to geographic region, institution type, and endourological training background

Stratification	Parameter	Main finding	*p* value
Endourology training background	Pre-stenting duration	Respondents with fellowship training more frequently preferred a 2-week pre-stenting duration, whereas those trained primarily during residency or through consultant practice more commonly favored durations of 3–4 weeks.	**0.042**
	Long-term stent exchange interval	3-month exchanges were more commonly preferred among residency-trained respondents, whereas 12-month intervals were favored by those with consultant practice–based experience.	**0.039**
	Imaging confirmation of stent position	Higher rates of routine imaging confirmation among residency-trained and consultant practice–trained respondents; significant linear trend across training backgrounds.	0.065*
Geographic region	Stenting after complicated URS	Routine stenting after complicated URS was more common in Europe (79.4%) and Asia-Pacific (77.8%) than in the Americas (66.7%) and the Middle East (54.2%)	0.073
	Stent-related symptom burden	Americas-based respondents most commonly reported stent-related symptoms in 25–50% of patients, whereas Middle East respondents more commonly reported symptom rates of 50–75%	0.081
	Preferred stent diameter for ureteral strictures	6 Fr stents predominated in the Americas (88.9%), whereas larger-caliber stents (7–8 Fr) were more common in Europe and the Middle East.	**0.002**
	Prescription of alpha-blockers/anticholinergics	Routine prescription was most common in the Americas, where 44.4% of respondents reported always prescribing these medications, whereas respondents from the Asia-Pacific region most frequently reported use in more than 50% of cases.	**0.048**
Institution type	Preferred stent diameter for pre-stenting	Stent diameter preferences for pre-stenting differed significantly according to institution type.	**0.015**
	Most commonly used stent diameter	6 Fr stents predominated in private hospitals, whereas larger-caliber stents were more common in university hospitals.	**0.015**
	Postoperative stenting after suction access sheath use	Routine postoperative stenting was more common in university hospitals, whereas public hospitals more frequently reported no stent placement.	**0.046**
	Dedicated stent follow-up protocol/clinic	Structured follow-up systems were more common in university hospitals (69.2%) than in public (43.5%) or private institutions (48.4%)	**0.038**

*Overall Pearson χ² p = 0.065, Linear-by-Linear Association p = 0.002

## Discussion

In this international survey of endourologists from 48 countries, we identified substantial variability in ureteral stent practices. The most notable findings were the persistent use of stents after uncomplicated URS, heterogeneous approaches to pre-stenting and pharmacologic management, and the limited adoption of structured stent-tracking systems and validated symptom-assessment tools, highlighting an important gap between contemporary evidence and real-world clinical practice.

Our survey demonstrated marked heterogeneity in pre-stenting practices. Although routine pre-stenting was uncommon, respondents most frequently used it following failed URS or to facilitate ureteral dilatation. Considerable variation was also observed regarding pre-stenting duration. This variability is noteworthy because recent evidence suggests that passive ureteral dilatation requires at least two weeks to achieve optimal outcomes, whereas prolonged dwell times may increase infectious complications [6, 7]. Stone-free rates appear lower when pre-stenting duration is shorter than two weeks, while durations beyond five weeks have been associated with increased perioperative morbidity and postoperative infections [6–8]. Interestingly, our subgroup analysis demonstrated that fellowship-trained respondents more frequently preferred a two-week pre-stenting duration, whereas those trained primarily during residency or consultant practice more commonly favored longer durations. Although exploratory, this finding suggests that differences in training background may influence the adoption of emerging evidence regarding optimal pre-stenting duration.

One of the most important findings of this survey was the continued high use of ureteral stents after uncomplicated URS. Despite contemporary EAU and AUA guideline recommendations supporting stent omission in carefully selected patients, a substantial proportion of respondents reported routine postoperative stenting. This observation is consistent with previous studies demonstrating that real-world practice has been slower to adopt stent-free URS than the available evidence would support. Randomized trials have shown that omission of stenting in selected uncomplicated cases reduces postoperative pain, irritative urinary symptoms, and analgesic requirements while maintaining comparable stone-free rates and low rates of clinically significant obstruction [4, 8]. Nevertheless, routine stenting remains common, likely reflecting concerns regarding postoperative complications, surgeon preference, medicolegal considerations, and limitations in postoperative follow-up systems.

Our survey also provided insight into stent use in special patient populations. In pregnant patients, impaired renal function, recurrent febrile urinary tract infection, and persistent pain were the most influential factors guiding stent placement. These findings are consistent with current evidence supporting ureteral stenting as an effective decompressive strategy during pregnancy, despite the increased risk of encrustation and stent-related complications in this population [9–11]. The indications reported by our respondents therefore appear to reflect a risk-based approach that balances maternal and fetal safety with the need for effective urinary drainage. Similarly, substantial variability was observed in pediatric stenting practices, particularly regarding stent size, length selection, and clinical indications. Most respondents preferred smaller-caliber stents and individualized stent lengths according to patient characteristics. These findings align with current evidence suggesting that pediatric ureteral stenting requires individualized decision-making because adult-derived strategies are not directly transferable to children [12].

Stent-related symptoms (SRSs) were highly prevalent in our survey, with urgency and frequency representing the most commonly reported complaints. This mirrors previous studies identifying lower urinary tract symptoms, pain, and hematuria as the principal contributors to reduced quality of life following stent placement [13–15]. Respondents also frequently reported encrustation, hematuria, flank pain, and urinary tract infection, consistent with the known morbidity associated with prolonged stent dwell times [16–18]. The use of extraction-string stents remained limited and was largely confined to short-term stenting, despite evidence supporting less painful stent removal and avoidance of cystoscopic extraction [17].

 Another important observation was the considerable variability in the pharmacologic management of stent-related symptoms. Although α-blockers and anticholinergic agents were commonly used, prescribing patterns varied substantially among respondents. This finding is notable because both EAU and AUA guidelines support pharmacologic therapy, particularly α-blockers, for reducing stent-related discomfort [8, 18]. Regional differences were also observed, suggesting that local practice patterns continue to influence symptom-management strategies despite broadly similar overall stenting practices. In parallel, the low utilization of hydrogel-coated, drug-eluting, metallic, and biodegradable stents observed in our survey indicates that newer stent technologies have not yet achieved widespread clinical adoption despite their theoretical advantages [19].

A particularly important finding of this survey relates to patient safety and follow-up. Although more than half of respondents reported having a dedicated follow-up protocol or clinic, nearly half did not use structured reminder or alert systems for stent tracking and removal. Furthermore, more than 70% had never used a validated symptom-assessment tool such as the Ureteral Stent Symptom Questionnaire (USSQ). These findings reveal a disconnect between the recognized burden of stent-related symptoms and the routine use of objective symptom assessment. The USSQ remains the most comprehensive validated instrument for evaluating stent-related quality of life, yet its implementation in everyday practice remains limited. Given the substantial clinical and economic consequences of forgotten stents, the limited adoption of tracking systems represents a potentially preventable patient safety gap.

Taken together, our findings suggest that future efforts should focus not only on determining when a stent should be placed but also on optimizing the entire stent pathway, including patient selection, dwell time, symptom monitoring, and follow-up systems. Despite ongoing advances in stent technology and pharmacologic management, substantial variability persists in everyday practice. Greater implementation of evidence-based recommendations, structured tracking systems, standardized symptom-assessment tools, and patient education strategies may represent the most achievable measures for reducing stent-related morbidity and improving patient safety in contemporary endourological practice.

### Limitations of the study

This study has several limitations. The survey-based and self-reported design may introduce response and selection bias, and the reported practices may not fully reflect real-world clinical practice. Although responses were obtained from 48 countries, the sample size remains limited and the findings should not be considered fully representative of worldwide ureteral stent practices. Because the survey was distributed through social media platforms, scientific meetings, professional networks, and the EAU Endourology Section, the total number of individuals reached could not be determined and a response rate could not be calculated. Respondents may also have been more academically active and more engaged in endourological practice than the broader urological community, potentially limiting generalizability. In addition, information regarding institutional case volume and exposure to specialized patient populations was not collected. Finally, subgroup analyses should be interpreted cautiously because they were exploratory, involved relatively small comparison groups, and were not adjusted for multiple comparisons.

## Conclusion

This international survey highlights substantial variability in contemporary ureteral stent practices despite the availability of guideline recommendations and growing evidence in several aspects of stent management. Routine stent placement remains common after uncomplicated URS, while approaches to pre-stenting, symptom management, and follow-up vary considerably among endourologists. Although core practices were largely comparable across geographic regions, differences according to training background and institution type suggest that local factors continue to influence clinical decision-making. Stent-related morbidity remains highly prevalent, with urinary symptoms and stent-related complications representing a significant burden in daily practice. Importantly, major gaps were identified in patient follow-up, including the limited use of stent-tracking systems and validated symptom-assessment tools. Future efforts should focus not only on appropriate stent utilization but also on optimizing the entire stent pathway through evidence-based practice, structured follow-up systems, routine symptom assessment, and improved patient education. Such measures may help reduce stent-related morbidity and improve patient safety worldwide.

## Data Availability

The datasets generated and/or analyzed during the current study are not publicly available due to privacy considerations but are available from the corresponding author on reasonable request.
